# Neuronal NTPDase3 Mediates Extracellular ATP Degradation in Trigeminal Nociceptive Pathway

**DOI:** 10.1371/journal.pone.0164028

**Published:** 2016-10-05

**Authors:** Lihua Ma, Thu Trinh, Yanfang Ren, Robert T. Dirksen, Xiuxin Liu

**Affiliations:** 1 Department of Dentistry, Eastman Institute for Oral Health, University of Rochester School of Medicine and Dentistry, Rochester, NY, United States of America; 2 Department of Dentistry, School of Stomatology, Zhengzhou University, Zhengzhou, China; 3 Department of Pharmacology and Physiology, University of Rochester School of Medicine and Dentistry, Rochester, NY, United States of America; Yeshiva University Albert Einstein College of Medicine, UNITED STATES

## Abstract

ATP induces pain via activation of purinergic receptors in nociceptive sensory nerves. ATP signaling is terminated by ATP hydrolysis mediated by cell surface-localized ecto-nucleotidases. Using enzymatic histochemical staining, we show that ecto-ATPase activity is present in mouse trigeminal nerves. Using immunofluorescence staining, we found that ecto-NTPDase3 is expressed in trigeminal nociceptive neurons and their projections to the brainstem. In addition, ecto-ATPase activity and ecto-NTPDase3 are also detected in the nociceptive outermost layer of the trigeminal subnucleus caudalis. Furthermore, we demonstrate that incubation with anti-NTPDase3 serum reduces extracellular ATP degradation in the nociceptive lamina of both the trigeminal subnucleus caudalis and the spinal cord dorsal horn. These results are consistent with neuronal NTPDase3 activity modulating pain signal transduction and transmission by affecting extracellular ATP hydrolysis within the trigeminal nociceptive pathway. Thus, disruption of trigeminal neuronal NTPDase3 expression and localization to presynaptic terminals during chronic inflammation, local constriction and injury may contribute to the pathogenesis of orofacial neuropathic pain.

## Introduction

Noxious stimuli or pain mediators released following tissue injury or inflammation activate nociceptors in peripheral sensory nerve fibers. Noxious stimulation of trigeminal nerves that innervate orofacial tissue results in transduction of the pain signal to secondary nociceptive neurons in the brainstem trigeminal subnucleus caudalis. Pain sensation also depends on the condition and status of the sensory nervous system. As sensitization occurs within the nociceptive signal pathway, severe pain is induced by slight noxious stimulation or even non-noxious stimulation. It is well established that ATP acts as a transmitter that participates in neuronal transmission in the nervous system [1]. ATP and its metabolites are also important pain mediators and modulators of pain signal processing in nociceptive sensory nerves [2–5]. Purinergic P2X receptors are expressed in trigeminal nerve fibers [6] and ATP induces pain by activation of P2X3 receptors in peripheral nerves [4]. ATP also participates in the process of pain sensitization by activating P2X3 receptors, as well as interaction with other neurotransmitters and modulators of nociceptive neurons [7] [8]. Recently, P2X7 receptors expressed in medullary microglia were shown to be involved in the process of central sensitization of neuropathic pain [9]. However, the integrated role of purinergic receptor signaling in trigeminal nerves in mediating orofacial neuropathic pain remains largely unknown.

Purinergic signaling depends on ATP release, purinergic receptor action and subsequent termination via ATP hydrolysis to ADP, AMP and adenosine [10]. Live cells contain high concentrations of ATP (mM), and thus, are capable of providing relatively large amounts of local ATP following tissue injury and inflammation. However, ecto-nucleotidases quickly hydrolyze extracellular ATP to ADP and AMP. AMP is further hydrolyzed to adenosine by ecto-5’-nucleotidase (CD73) and to a lesser extent by a transmembrane isoform of prostatic acid phosphatase (PAP) [11] [12]. ATP and its metabolites mediate different cellular effects via activation of purinergic ionotropic P2X receptors, metabotropic P2Y, and P1 receptors [13]. For example, ATP usually activates P2X receptors to induce pain in peripheral nerves [3,14], while adenosine mediates analgesia via activation of A1 receptors [15]. Thus, ecto-nucleotidases affect nociception by terminating ATP-induced pain transduction and promoting adenosine-mediated analgesia. Because of their dynamic catalytic activities under physiological conditions, ecto-nucleoside triphosphate diphosphohydrolases (NTPDases) are the dominant enzymes involved in hydrolyzing extracellular ATP and ADP [12,16]. Three members of the ecto-NTPDase family (i.e. NTPDase1, NTPDase2 and NTPDase3) are expressed in the nervous system [17]. NTPDase1 and NTPDase3 hydrolyze both ATP and ADP, while NTPDase2 primarily hydrolyzes ATP with minimal ADP hydrolytic activity [12]. Since ATP and its metabolites participate in pain signal processing via activation of purinergic P2X, P2Y or A1 receptors, identification of the expression pattern and activity of different ecto-nucleotidases in the nociceptive nervous system is necessary to fully understand the precise role of purinergic signaling in nociception.

## Materials and Methods

### Sample preparation

All animal experiments were approved by the University Committee on Animal Resources (UCAR) at the University of Rochester. Trigeminal ganglia (TG), trigeminal nerve trunks, spinal cords and brainstems were obtained from 11 male and female WT C57Bl6 mice (4–6 month). The animals were maintained in the University Vivarium under a natural daylight cycle, with food and water ad libitum. Briefly, after anesthesia with intraperitoneal injection of ketamine (100mg/Kg) and xylazine (10mg/Kg), animals were perfused with ice cold 1X PBS and then 4% paraformaldehyde (4°C) for 10 min. TG, brainstems and spinal cords were subsequently isolated from the skull or spine. Trigeminal nerve trunks that project to the medulla were also collected. For histochemical examination, TG, spinal cord and brainstem blocks or TG nerve trunks were sectioned to 25μm thick slices using a cryostat microtome (Leica-2000) and then collated on gelatin-coated microscope slides. Sections were then stored at -20°C until future use. For immunofluorescence staining, samples were post-fixed for 12 hours with 4% paraformaldehyde in 0.1 M phosphate-buffered saline (PBS) and then dehydrated in 30% sucrose for 12 hours. 20μm thick sections were made using a cryostat microtome and mounted on gelatin-coated microscope slides.

### Immunofluorescence staining

Prepared TG, trigeminal nerve trunk and brainstem sections were first incubated with blocking solution (1% bovine serum albumin (BSA)/1% normal goat serum/0.04% Triton X-100 in 1X PBS) for 2 hours at 4°C and then incubated overnight with antibodies against different antigens in incubation solution (1% BSA, 1% normal goat serum in 1X PBS). The primary antibodies used in this study include mouse anti-MAP2 (1:1000, Sigma), mouse anti-human NTPDase3 (hN3-H10s, 1:1000), guinea pig anti-mouse NTPDase1 (mN1-2c, 1:500), rabbit anti-mouse NTPDase2 (mN2-35l, 1:500), and guinea pig anti-mouse NTPDase3 (mN3-3c, 1:500). All NTPDase antibodies were kindly provided by Dr. Sevigny at the University Laval (www.ectonucleotidases-ab.com), their specificities have been previously characterized [18–20]. For IB4 co-staining, Fluorescein isothiocyanate (FITC) conjugated Isolectin B4 (Bandeiraea simplicifolia, Enzo, 1:400) was included in the incubation solution. After washing with 1X PBS, sections were incubated for 2 hours with secondary antibodies conjugated with either Alexa Fluor 488 or Alexa Fluor 543 (Molecular Probes, 1:200). Sections were then rinsed with 1X PBS and covered in mounting medium with a cover slip. In some slides, sections were co-stained with Hoechst (1mg/ml) for cell nucleus identification. Control experiments were performed using the same protocol with the absence of pre-immune serum. Sections were viewed and captured using a confocal microscope (Olympus, Japan).

### Nucleotidase activity histochemistry

To demonstrate functional ecto-ATPase activity in TG, brainstem and spinal cord slices, a lead phosphate method previously described was employed [21]. In brief, frozen sections were warmed to room temperature and fixed with 4% paraformaldehyde in PBS for 30 min. After washing with 1X PBS, sections were preincubated for 30 min at room temperature with a Tris-maleate-sucrose buffer (0.25 M sucrose, 50 mM Tris-maleate, pH 7.4) containing 2 mM CaCl_2_. The enzyme reaction was performed for 60 min at room temperature in a Tris-maleate-sucrose-buffered substrate solution (2 mM Pb(NO_3_)_2_, 5 mM MnCl_2_, 2 mM CaCl_2_, 50 mM Tris-maleate, pH 7.4, plus 0.25 M sucrose and 3% dextran T250 (Roth, Karlsruhe, Germany)) containing 1 mM ATP (sodium salt, Sigma) as substrate. After washing with demineralized water, the sections were incubated in an aqueous solution of (NH_4_)_2_S (1% v/v) for 30s. Lead orthophosphate precipitated as a result of nucleotidase-mediated ATP hydrolysis was visualized as a brown deposit. Subsequently, the sections were dehydrated in graded ethanol and mounted with Permount mounting medium (Fisher Scientific). To account for nonspecific phosphatase activity, sections were incubated with para-nitrophenyl phosphate (1 mM; Sigma) as a substrate. In some experiments, levamisole (Sigma,10 mM) and ouabain (Sigma, 5 mM) were included to inhibit alkaline phosphatase and sodium/potassium (Na^+^/K^+^)-ATPase, respectively. To control for non-specific lead precipitation, control experiments were conducted in the absence of substrate in the incubation solution. Under these experimental conditions (pH = 7.4), this assay reports the aggregate activity of all nucleotidases (NTPDase1, 2, 3 and 8) [12].

### Anti-NTPDase3 serum incubation and quantification of ecto-ATPase activity

To demonstrate that ecto-NTPDase3 is responsible for ecto-ATPase activity detected in nociceptive lamina of the spinal cord or brainstem, we tested if incubation with a specific anti-NTPDase3 antibody could reduce the ecto-ATPase activity in spinal cord dorsal horn or trigeminal subnucleus caudalis. For these experiments, continuous sections (25μm) from the same spinal cord or brainstem were collected. Two adjacent sections were mounted side by side on the same slide. Before nucleotidase histochemical staining, one section was incubated in incubation solution (1X PBS solution containing 0.04% triton, 1% BSA and 1% normal goat serum) with guinea pig anti-mouse NTPDase3 serum (1:100) for 24 hours at 4°C. The other section was incubated in incubation solution with either normal guinea pig serum or same animal pre-immunization serum (1:100) as control. After rinsing in NS, both sections were then used for nucleotidase histochemical staining as described above. Histochemical staining was assessed using an Olympus microscope and pictures captured using a digital camera (Moticam-1000).

To quantify relative ecto-ATPase activity, average staining intensity in the region of nociceptive lamina of dorsal horn or trigeminal subnucleus caudalis was obtained using Image J, and then normalized by the background intensity. As an internal control, ecto-ATPase activity staining intensity within the region of large blood vessels was also obtained. The difference in staining intensity between sections incubated with control serum and anti-NTPDase3 serum was quantified as means ± S.E. and analyzed using unpaired student’s t-test with p<0.05 considered as statistically significant. “n” values represent the number of slices observed in each group.

## Results

### Detection of functional ecto-ATPase activity in trigeminal nerves

**Fig 1:**
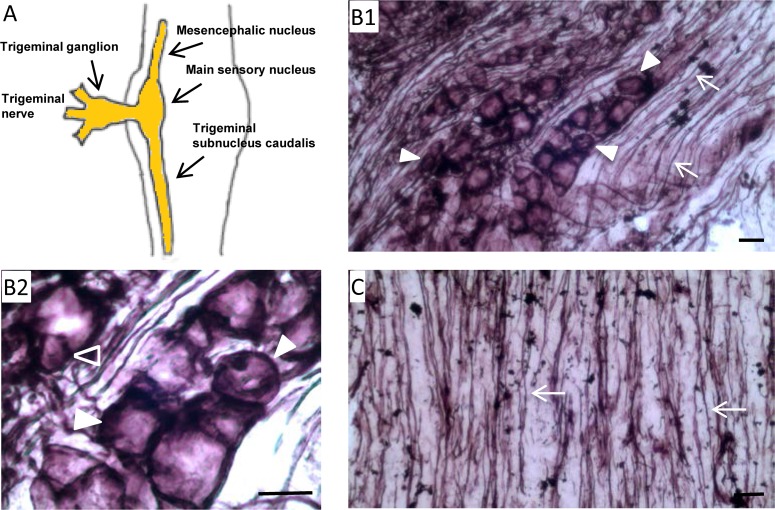
Detection of ecto-ATPase activity in trigeminal nerves. A: Diagram illustrating the trigeminal nerve system. B: Histochemical staining for ecto-ATPase activity in trigeminal ganglia. B1: Ecto-ATPase activity is detected in both trigeminal ganglia (TG) neurons (solid triangles) as well as in nerve fibers (arrows); B2: Ecto-ATPase activity is detected in both large (solid triangles) and small (open triangle) TG neurons. C: Histochemical staining for ecto-ATPase activity in trigeminal nerves trunks. Ecto-ATPase activity is detected in trigeminal nerve fibers projecting to the brainstem (arrows). Scale bars: 10 μm for B1, B2 and C respectively.

Purinergic signaling modulates nociception transduction in the sensory nerve system. Our previous work found that NTPDase2 is expressed in satellite cells and Schwann cells in trigeminal nerves [18]. Here, we tested for functional ecto-ATPase activity in trigeminal nerves (Fig 1A). As shown in Fig 1B, positive staining for ecto-ATPase activity was detected in TG sections. Specifically, ecto-ATPase activity was detected in presumptive TG neurons (Fig 1B1 and 1B2), as well as in their corresponding nerve fibers (Fig 1B1). In addition, we also detected positive staining for functional ecto-ATPase activity in trigeminal nerve fibers projecting to the brainstem (Fig 1C). Detection of functional ecto-ATPase activity in presumptive TG neurons and nerve fibers suggests the expression of ecto-nucleotidases in trigeminal neurons.

### Expression of ecto-NTPDase3 in TG neurons

**Fig 2:**
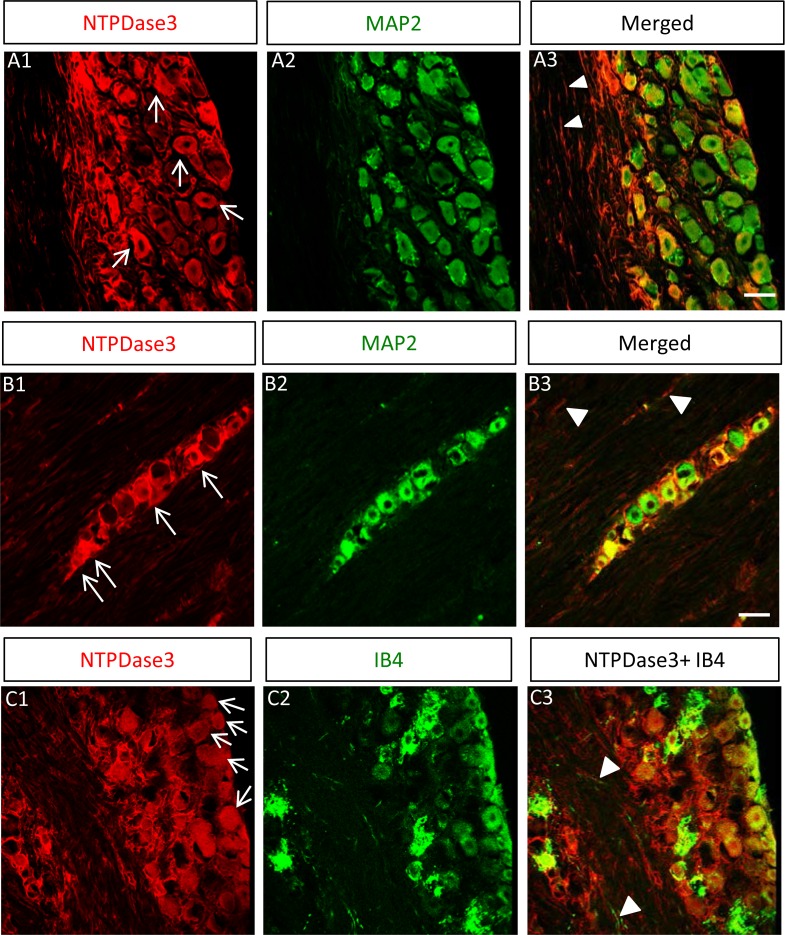
Ecto-NTPDase3 is expressed in TG neurons. A: Ecto-NTPDase3 is detected in TG neurons. Immunostaining for NTPDase3 (A1) is co-localized with the neuronal marker MAP2 (A2) in TG neurons (arrows in A1) and their axonal fibers (white triangles in merged image in A3. B: Ecto-NTPDase3 is detected in trigeminal nerve trunks projecting to the brainstem. Immunostaining for NTPDase3 (B1) is co-localized with the neuronal marker MAP2 (B2) in nerve fibers (white triangles in merged image in B3) as well as in a cluster of TG neurons (arrows in B1) within the nerve trunk projecting to the brainstem. C: Ecto-NTPDase3 is detected in TG nociceptive neurons. Immunostaining for NTPDase3 (C1) is co-localized with the nociceptive neuronal marker IB4 (C2) in TG nociceptive neurons (arrows in C1) and their axonal nerve fibers (white triangles in merged image in C3). Scale bars: 15, 15 and 15μm for A-C, respectively.

Three ecto-nucleotidases (NTPDase1, 2 and 3) are expressed within the nervous system [22,23]. Therefore, we tested for subtype-specific ecto-NTPDase expression in TG neurons. As shown in Fig 2A, positive immunoreactivity staining for ecto-NTPDase3 was detected in TG cells. To confirm ecto-NTPDase3 expression specifically in TG neurons, co-immunostaining with neuronal marker MAP2 was performed. Consistent with expression in TG neurons, all NTPDase3-positive cells (100%) co-localized with MAP2 staining (a total of 103 NTPDase3-positive cells were identified in 4 different slices, Fig 2A). In addition to expression of ecto-NTPDase3 in TG neurons, we also noted ecto-NTPDase3 expression in a cluster of TG neurons within nerve trunks that project to the brainstem (Fig 2B). The expression of NTPDase3 in TG neurons was not homogeneous, but exhibited mosaicism. Specifically, while some TG neurons displayed strong NTPDase3 staining (Fig 2A and 2B), others displayed weak (Fig 2A and 2B) or even lacked NTPDase3 staining (Fig 2A and 2B). The percentages of TG neurons exhibiting strong, weak or negative NTPDase3 staining was 26%, 55% and 19%, respectively (a total of 260 MAP2 positive neurons were identified in 7 different slices). In addition, we also observed positive staining for NTPDase3 in axonal nerve fibers within the TG (Fig 2A) and in nerve trunks projecting to the brainstem (Fig 2B).

To demonstrate that NTPDase3 is expressed in TG nociceptive neurons, we co-stained with the nociceptive neuronal marker IB4. As demonstrated in Fig 2C, most IB4 positive neurons exhibited robust NTPDase3 staining (95% of 74 IB4 positive neurons identified in 3 slices), while only a few IB4 positive neurons were negative for NTPDase3 staining. We also noted that some NTPDase3-positive neurons did not stain for IB4 or displayed weak IB4 staining (Fig 2C). Since only a fraction of all TG nociceptive neurons are IB4 positive [24], the nociceptive identity in these IB4-negative, NTPDase3-positive neurons needs to be determined. We further tested for the expression of other ecto-NTPDase isoforms in TG neurons. While we confirmed expression of NTPDase2 in satellite cells and NTPDase1 in blood vessels as previously reported [18], ecto-NTPDase8 was not detected in TG neurons (data not shown).

### Detection of ecto-ATPase activity and ecto-NTPDase3 in trigeminal subnucleus caudalis

**Fig 3:**
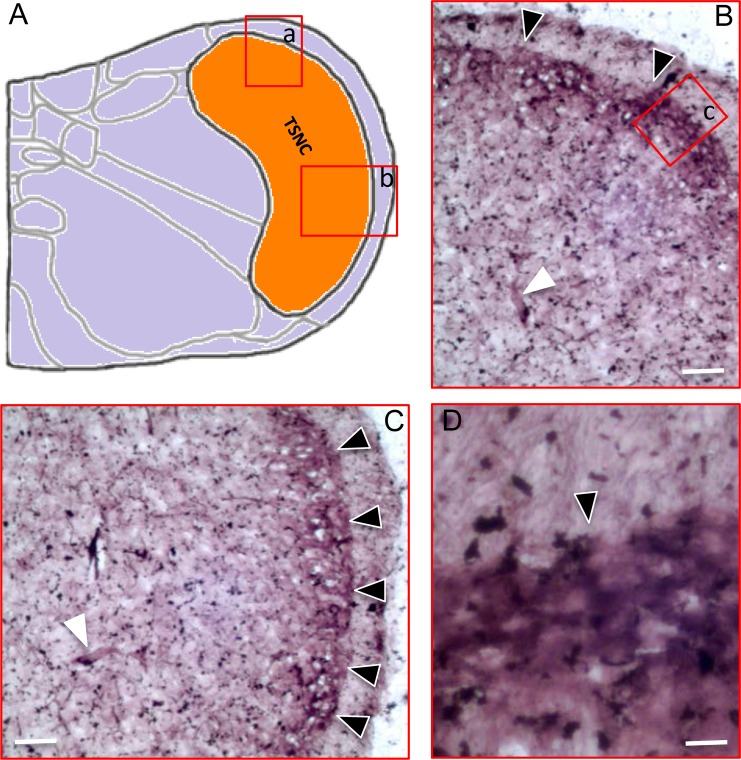
Detection of ecto-ATPase activity in the trigeminal subnucleus caudalis of the brainstem. A: Diagram illustrating the trigeminal subnucleus caudalis (TSNC) of the brainstem. B: Ecto-ATPase activity is detected in the trigeminal subnucleus caudalis corresponding to the region (a) marked in A. C: Ecto-ATPase activity in the trigeminal subnucleus caudalis corresponding to the region (b) marked in A. D: Ecto-ATPase activity in the corresponding region (c) marked in B. Note: For panels B-C, ecto-ATPase activity is mainly located at the outermost layer of the trigeminal subnucleus caudalis (black triangles). Ecto-ATPase activity is also detected in large blood vessels in the brainstem (white triangles). Scale bars: 50, 50 and 20μm for B-D respectively.

The primary nociceptive neurons in the TG and dorsal root ganglia send axonal fibers to form synapses with secondary nociceptive neurons in the brainstem and spinal cord. It is well established that nociceptive Aσ fibers and C fibers project to the nociceptive outermost layers (lamina I/II) of the dorsal horn. However, it is not clear if ecto-NTPDase3, expressed in TG nociceptive neurons, is also detectable in the nociceptive lamina of the trigeminal subnucleus caudalis in the brainstem (Fig 3A). Therefore, we first assessed functional ecto-ATPase activity in the brainstem. As illustrated in Fig 3B–3D, ecto-ATPase activity was detected in the outermost layer of the trigeminal subnucleus caudalis, which corresponds to the nociceptive lamina (I/II) of the spinal cord dorsal horn.

**Fig 4:**
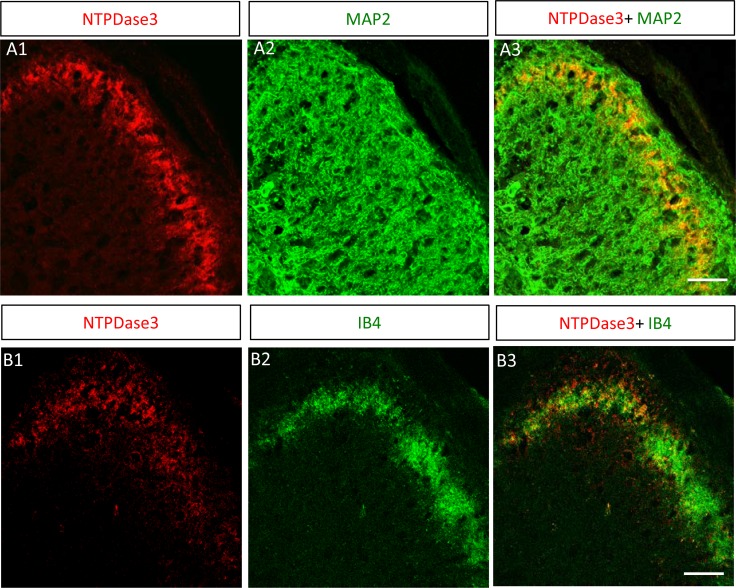
Expression of ecto-NTPDase3 in trigeminal subnucleus caudalis. A: Ecto-NTPDase3 is expressed in the outermost layer of the trigeminal subnucleus caudalis. A1: Immunostaining for ecto-NTPDase3 is detected in the brainstem at the lower level of the trigeminal subnucleus caudalis. A2: Co-staining with neuronal marker MAP2 shows the outline of the trigeminal subnucleus caudalis. A3: Merged image highlights that NTPDase3 is expressed within the outermost layer of the trigeminal subnucleus caudalis. Scale bar: 40μm. B: Ecto-NTPDase3 is expressed in the nociceptive lamina of the trigeminal subnucleus caudalis. B1: Immunostaining of ecto-NTPDase3 in the brainstem at the lower level of the trigeminal subnucleus caudalis. B2: Co-staining with the nociceptive neuronal marker IB4 in the brainstem demonstrates the location of the nociceptive lamina of the trigeminal subnucleus caudalis. B3: Merged image of ecto-NTPDase3 and IB4 staining shows that NTPDase3 is expressed in the nociceptive lamina of the trigeminal subnucleus caudalis. Scale bar: 50μm.

We next used immunostaining to confirm the presence of ecto-NTPDase3 in the trigeminal subnucleus caudalis. As expected, positive immunostaining for ecto-NTPDase3 was detected in the brainstem (Fig 4A1). By double immunostaining with MAP2 (Fig 4A2), we also demonstrated that NTPDase3 is expressed in the outermost layer of the trigeminal subnucleus caudalis (Fig 4A3). The immunostaining pattern of NTPDase3 was characterized by a plaque or punctate distribution, consistent with NTPDase3 in trigeminal subnucleus caudalis being localized primarily at presynaptic terminals.

To confirm the presence of NTPDse3 in nociceptive lamina of the trigeminal subnucleus caudalis, we co-stained with the nociceptive neuronal marker IB4. NTPDase3 staining overlapped with IB4 staining within the nociceptive outermost layers of the trigeminal subnucleus caudalis (Fig 4B), which corresponded to the nociceptive lamina (I/II) of the spinal cord dorsal horn. We also examined the expression of other ecto-nucleotidases (NTPDase1, 2 and 8), but none of these were detected in the nociceptive lamina of the trigeminal subnucleus caudalis (data not shown). The presence of ecto-ATPase activity and NTPDase3 expression in the nociceptive lamina of the trigeminal subnucleus caudalis suggests a potential role of ATP signaling in pain signal transmission and/or modulation in the brainstem.

### Incubation with anti-NTPDase3 serum reduces extracellular ATP degradation in the nociceptive lamina

**Fig 5:**
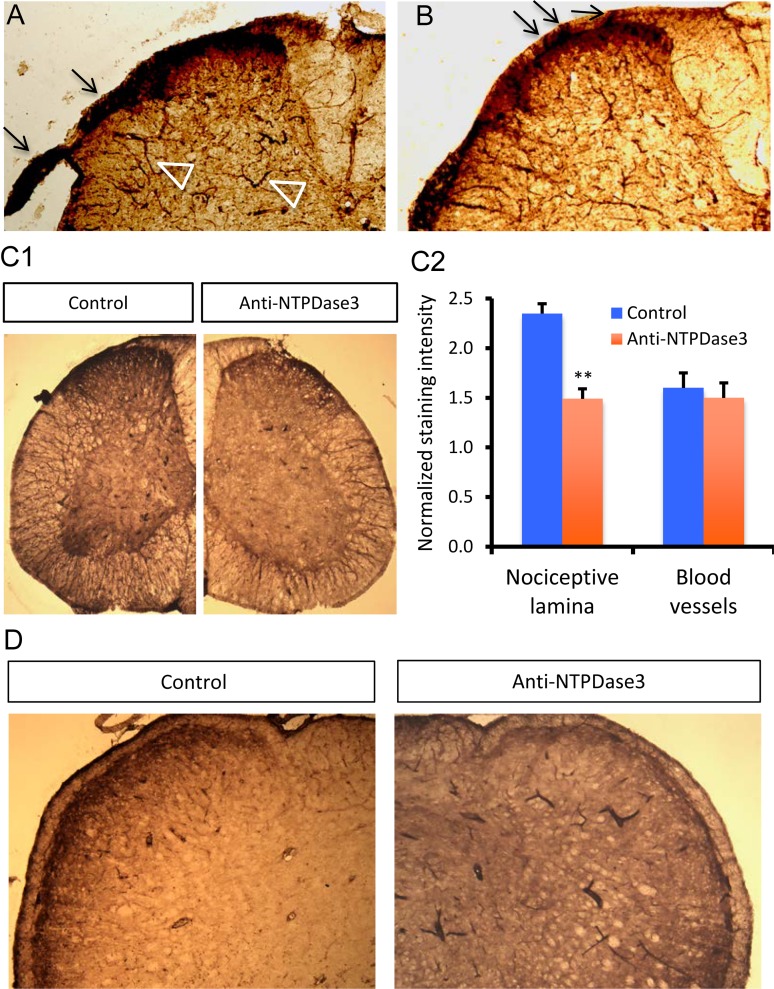
Ecto-NTPDase3 mediates ATP degradation in the nociceptive lamina of the spinal cord dorsal horn and trigeminal subnucleus caudalis. A: Enzymatic histochemistry detection of ecto-ATPase activity in the spinal cord. Positive staining for ecto-ATPase activity is detected in dorsal root nerve (arrows) and in the outermost layer (nociceptive lamina) of the dorsal horn. Ecto-ATPase activity is also detected in large blood vessels (open triangles) in the spinal cord. B: Ecto-ATPase activity is detected in dorsal root nerve fibers that project to the nociceptive lamina of the dorsal horn. Arrows indicate the positive ecto-ATPase activity staining in dorsal root nerve fibers that penetrate the surface of the spinal cord and project to the nociceptive lamina of the dorsal horn. C: Pre-incubation with specific anti-NTPDase3 serum reduces ecto-ATPase activity staining in the spinal cord dorsal horn. C1: Ecto-ATPase activity staining in spinal cord sections incubated with either control serum (left) or anti-NTPDase3 serum (right). The intensity of ecto-ATPase activity staining in the dorsal horn nociceptive lamina is reduced after incubation with anti-NTPDase3 serum. C2: Changes of normalized ecto-ATPase activity staining intensity in sections after incubation with either control or anti-NTPDase3 serum. The staining intensity for ecto-ATPase activity either in the dorsal horn nociceptive lamina or blood vessels was normalized by the background insensitivity. Unpaired student’s t-test, n = 4, **p<0.01. D: Pre-incubation with anti-NTPDase3 serum reduces the ecto-ATPase activity staining in nociceptive outermost layer of the trigeminal subnucleus caudalis. Enzymatic histochemical staining for ecto-ATPase activity in sections after incubation with either control serum (left) or anti-NTPDase3 serum (right). The intensity of ecto-ATPase activity staining in nociceptive outermost layer of the trigeminal subnucleus caudalis is reduced after incubation with anti-NTPDase3 serum. Note the intensity of ecto-ATPase activity staining in large blood vessels is not reduced, but is even enhanced after incubation with anti-NTPDase3 serum.

Expression of NTPDase3 in TG nociceptive neurons and the observed NTPDase3 staining pattern in trigeminal subnucleus caudalis suggests that NTPDase3 is likely synthesized in primary nociceptive neurons and then transported to the presynaptic terminals where it hydrolyzes synaptic ATP. Using histochemical staining, we evaluated NTPDase activity within dorsal root nerve fibers projecting to the spinal cord dorsal horn. As illustrated in Fig 5A, positive ecto-ATPase activity staining within the dorsal root nerve trunk was identified under control conditions. Fig 5B demonstrates that the positive staining penetrated the surface of the spinal cord and projected into the outermost layers of the dorsal horn. These observations support the transportation of NTPDase3 down TG nerve fibers to presynaptic terminals located at the central nervous nociceptive lamina.

To demonstrate that ecto-NTPDase3 is responsible for extracellular ATP degradation in the central nervous nociceptive lamina, we tested if incubation with a specific anti-NTPDase3 antibody reduced eco-ATPase activity in the central nervous nociceptive lamina. As shown in Fig 5C1, incubation with anti-NTPDase3 serum reduced the ecto-ATPase activity staining in the outermost layer of spinal cord dorsal horn compared to that observed under control conditions. In contrast, ecto-ATPase activity staining in large blood vessels was less affected by incubations with anti-NTPDase3 serum, consistent with the observation that NTPDase1 is expressed in blood vessels [25]. Quantification of the normalized staining intensity confirmed a statistically significant reduction in normalized staining confined within the nociceptive lamina (2.35 ± 0.10 vs. 1.49 ± 0.12, n = 4, p<0.01), but not in blood vessels (1.60 ± 0.15 vs. 1.50 ± 0.12, n = 4) after incubation with anti-NTPDase3 serum (Fig 5C2). We also tested the blocking effect of anti-NTPDase3 serum on ecto-ATPase activity staining in the brainstem. As expected, incubation with anti-NTPDase3 serum also significantly reduced normalized ecto-ATPase activity staining intensity in nociceptive lamina of trigeminal subnucleus caudlis compared to that observed under control conditions (Fig 5D, 2.80 ± 0.20 (n = 5) vs. 1.40 ± 0.20 (n = 6) for control and anti-NTPDase3 serum, respectively; p<0.01). In contrast, the intensity of normalized ecto-ATPase activity staining in large blood vessels, which express NTPDase1, is not reduced in sections pre-incubated with anti-NTPDase3 serum (Fig 5D). These results support the notion that neuronal ecto-NTPDase3 hydrolyzes extracellular ATP in the nociceptive lamina of the sensory nervous system.

## Discussion

Extracellular ATP activates P2X receptors and induces pain in peripheral sensory nerve fibers [3,4,14]. Recent evidence indicates that ATP and its metabolites, including adenosine, also affect pain signal transmission, modulation, inhibition and sensitization [7] in the central nervous nociceptive pathway. Depending on the availability of purinergic receptors, as well as ecto-nucleotidases responsible for extracellular ATP hydrolysis, ATP and its metabolites may differentially induce, modulate and mitigate pain by activating distinct ionotropic P2X receptors (permeable for Na^+^, K^+^, and Ca^2+^), metabotropic P2Y, or adenosine receptors.

Among the ecto-NTPDases in the nervous system [22,23], NTPDase1 and NTPDase3 hydrolyze both ATP and ADP, while NTPDase2 hydrolyzes primarily ATP with minimal activity for ADP [26]. Thus, NTPDase1 and NTPDase3 activity results in increases in AMP levels, which subsequently lead to increases in adenosine following hydrolysis by ecto-AMPases [11] [12]. On the other hand, NTPDase2 is primarily responsible for the production of extracellular ADP, which is an endogenous P2Y receptor agonist [22]. Our previous work revealed that NTPDase2 is expressed in Schwann cells and satellite cells associated with trigeminal nerves [18]. In this study, we extended this work to demonstrate that NTPDase3 is expressed in trigeminal ganglia nociceptive neurons and their axons. These results are consistent with NTPDase3 being detected in primary nociceptive neurons in the dorsal root ganglia [11,27,28] and trigeminal peripheral nerve Raschkow’s plexus in dental pulp [18]. We further confirmed functional ecto-ATPase activity in trigeminal ganglia neurons and associated nerve fibers that project to the brainstem and inhibition by anti-NTPDase3 serum. These results are consistent with a potential role of NTPDase3 in trigeminal nerve nociception by affecting the duration of extracellular ATP signaling. Since Aδ and C fibers essentially have thinner or lack myelin sheath derived from Schwann cells that express NTPDase2, the presence of functional NTPDase3 in trigeminal nociceptive neurons and their axonal fibers provides an alternative mechanism to terminate the pain signal in these neurons by reducing the concentration of ATP available to activate nociceptive P2X3 receptors [8]. In addition, NTPDase3 activity also provides the substrate (AMP) for generation of adenosine, which mediates both the peripheral and central analgesia [15].

The presence of NTPDase3 expression and ecto-ATPase activity within the outermost layer of trigeminal subnucleus caudalis, which corresponds to the nociceptive lamina (I/II) of the spinal cord dorsal horn, further extends the potential role of NTPDase3 in pain signal processing and transmission in trigeminal nociceptive nerves. Our results observed in the brainstem and other observations in spinal cord dorsal horn [25] suggest that NTPase3 in central nervous nociceptive lamina is likely synthesized in primary nociceptive ganglia neurons, and then transported to the presynaptic terminals via axons projecting to the secondary nociceptive neurons. However, the possibility of local translation of NTPase3 from mRNA at presynaptic terminals cannot be excluded. Nociceptive axonal terminals (C fiber and Aσ fibers) that contain peptide neuronal transmitter vesicles (CGRP, Substance P) are packaged in synaptic vesicles containing large amounts of ATP [29]. P2X receptors are highly sensitive to soluble factors like neuropeptides, neurotrophins, and are controlled by protein-protein interactions and discrete membrane compartmentalization [7]. Thus, ATP released from presynaptic vesicles could act as a neurotransmitter or modulator of pain signal transmission either by activation of the postsynaptic purinergic receptors or by interaction with co-packaged neuropeptide transmitters. By controlling the degradation of ATP within the synapse, NTPDase3 in presynaptic terminals is expected to reduce the amplitude and duration of pain signal transmission. However, NTPDase3 knock out mice display normal ecto-ATPase activity in the nociceptive lamina and do not show altered nociceptive behavior [27], suggesting that expression of alternative ecto-ATPases may be present or upregulated to compensate for the absence of NTPDase3 in the knock out mice. Interestingly, our ecto-ATPase activity blocking experiment showed that incubation with anti-NTPDase3 serum only partially reduced the ecto-ATPase activity in the nociceptive lamina. While these results indicate that NTPDase3 indeed mediates extracellular ATP degradation in trigeminal nociceptive lamina, they also leave open the possible role of alternative ecto-ATPases and illustrate the complexity of ATP hydrolysis mechanisms in the nociceptive lamina. Thus, a more definitive and comprehensive dissection for the possible ecto-ATPases responsible for extracellular ATP degradation in the central nervous nociceptive lamina waits further exploration.

Central sensitization occurs in patients presented with hyperalgesia and allodynia, which is usually accompanied with neuronal plasticity changes in nociceptive circuits [30]. Besides directly depolarizing secondary nociceptive neurons by activation of P2X receptors [7,9], ATP and its metabolites may also regulate central sensitization by affecting presynaptic vesicle release, postsynaptic receptor expression and/or neurotransmitter reuptake via both ionotropic and metabotropic purinergic receptors [7,31,32]. Indeed, purinergic receptors including P2X, P2Y, and adenosine receptors are expressed in the spinal cord dorsal horn [33]. Recently, P2X3 receptors were shown to interact with the synaptic scaffold protein calcium/calmodulin dependent serine protein kinase (CASK) and that activation of P2X3 receptors within the CASK/P2X3 complex contributes to neuronal plasticity and the release of neuromodulators and neurotransmitters [7]. ATP may also impact pain sensitization by affecting adjacent astrocytes and microglia cells in the nervous system. Activated microglia located in the nociceptive lamina express P2X4, P2X7 and P2Y12 receptors and activation of these receptors are involved in pathogenesis of neuropathic pain [9,34,35]. Therefore, the presence of NTPDase3 in the nociceptive lamina region could affect pain sensitization by modulating the purinergic receptor signaling in astrocytes and microglial cells. Reducing NTPDase3 within the brainstem and spinal cord dorsal horn would be expected to enhance pain transmission and induce pain sensitization by increasing the local concentration of ATP. Therefore, clinical conditions that disrupt NTPDase3 expression within TG nociceptive neurons and localization to peripheral and central axonal terminals may affect the pain signal transduction, transmission, conduction, modulation and sensitization in the trigeminal nociceptive nervous system.

## Conclusion

In this study, we identified the expression of ecto-NTPDase3 in trigeminal nociceptive neurons and demonstrated the presence of functional ecto-ATPase activity in trigeminal nerves. We further established that ecto-NTPDase3 mediates extracellular ATP degradation in the nociceptive lamina on the brainstem. By controlling the rates of ATP degradation and AMP generation, the primary substrate for adenosine production, neuronal NTPDase3 might play an important role in pain signal generation and modulation in trigeminal nociceptive nerves.
